# A new approach on assessing clinical pharmacists’ impact on prescribing errors in a surgical intensive care unit

**DOI:** 10.1007/s11096-019-00874-8

**Published:** 2019-07-22

**Authors:** Nora Kessemeier, Damaris Meyn, Michael Hoeckel, Joerg Reitze, Carsten Culmsee, Michael Tryba

**Affiliations:** 1 Gesundheit Nordhessen Holding AG - Pharmacy, Moenchebergstr. 41–43, 34125 Kassel, Germany; 2 Faculty of Pharmacy, Institute of Pharmacology and Clinical Pharmacy, University of Marburg, Karl-von-Frisch Str. 1, 35032 Marburg, Germany; 3 MoreDATA GmbH, Kerkrader Str. 11, 35394 Giessen, Germany; 4 Kassel School of Medicine, Moenchebergstr. 41–43, 34125 Kassel, Germany

**Keywords:** Clinical pharmacist, Germany, Intensive care unit, Medication error, Medication safety, Medication review, Prescribing error rate, Pharmacists interventions

## Abstract

*Background* With a clinical pharmacists’ participation in an intensive care unit (ICU) previous international studies have shown a reduction of medication errors, drug costs and improvements of clinical outcomes. Still there is a lack of qualitative data on clinical pharmacists’ impact on prescribing error rates in the ICU. Therefore, a new approach was developed relating prescribing errors to the number of monitored medications including physicians’ approval on all prescribing errors. *Objective* This study investigates the influence of clinical pharmacists’ medication review on the prescribing error rate in an ICU. *Setting* A controlled interventional study was conducted in a surgical ICU with one control phase (P0) and two intervention phases (P1 and P2). *Method* The investigation aimed to determine if the medication review by clinical pharmacists results in a significant reduction of prescribing errors related to a control period. In contrast to previous studies, prescribing errors detected by the clinical pharmacists, were only taken into account, if consent with the physicians was achieved. Secondary outcomes were the reduction of potentially severe prescribing errors, the number of days without systemic anti-infective therapy and the ICU length of stay. Throughout P0 the data was collected retrospectively without any intervention. During the intervention periods P1 and P2, two clinical pharmacists screened the medical records for prescribing errors and discussed them with the senior physician in charge. During P2 one clinical pharmacist attended ward rounds additionally. *Main Outcome Measure* The main outcome measure of this study was the number of prescribing errors detected related to the number of monitored medications. *Results* The incidence of prescribing errors was significantly reduced from 1660 in P0 to 622 in P1 respectively 401 in P2 (P0 vs. P1/P2 respectively; both *p* < 0.001; Fisher’s Exact Test) in total, respective 14.12% in P0 vs. 5.13% in P1 and 3.25% in P2 related to the monitored medications (P0:11755; P1:12134; P2:12329). *Conclusion* Clinical pharmacists’ interventions led to a significant reduction of prescribing errors in the ICU, contributing to a safer medication process. We strongly recommend a broad implementation of clinical pharmacists in ICUs.

**Electronic supplementary material:**

The online version of this article (10.1007/s11096-019-00874-8) contains supplementary material, which is available to authorized users.

## Impact on practice

- The implementation of a clinical pharmacist in the ICU team may help to reduce clinically important complications.
- Both pharmacists and physicians must be encouraged to further expand their cooperation on clinical wards.
- By implementing clinical pharmacists in an ICU, hospitals increase usage of the resources and skills of the clinical pharmacists’ for the sake of patient safety, whichwould otherwise lie idle.
- Despite the country-specific differences in work-tasks and education, the impact of German clinical pharmacists’ on patient safety is comparable to that of international clinical pharmacy services.

## Introduction

The benefit of having a clinical pharmacist (CP) in the intensive care unit (ICU) has been shown by numerous international studies. Previous investigations have found reductions in the number of medication errors, number of preventable adverse drug events and drug costs and an improvement in clinical outcomes such as the ICU length of stay (LOS) and mortality [1–10]. Nevertheless, there is a need for additional and improved evidence regarding the impact of CPs on medication error rates in an ICU as a patient safety outcome. Most studies present descriptive results or relate the detected medication errors to monitored patient days and not to the amount of medications prescribed. Methodical improvements are needed to generate more conclusive findings. In addition, valid data on the overall incidence of medication errors and the number, impact and acceptance of pharmaceutical interventions in German ICUs is very limited [11–13]. Due to this scarcity of data, a controlled interventional study was designed to investigate the benefits of having CPs in the ICU and their impact on the prescribing error (PE) rate, especially related to monitored medications. The new approach of the present study was to generate valid data on PE rates related to monitored medications as a patient safety outcome. In addition, to generate clinically relevant results, all identified PEs were confirmed by a physician before they were included.

### Aim of the study

The primary hypothesis of this study was that a medication review by CPs resulted in a significant reduction of PEs compared to the number of PEs in a control group in which CPs did not review the medication.

As a secondary hypothesis, we assumed that CPs’ on-ward participation would result in a significant reduction in the subgroup of potentially severe PEs and increases the number of days without systemic anti-infective therapy compared to those in a control group.

### Ethics approval

Approval from the Ethics Commission of the State Chamber of Physicians in Hesse was obtained (F132/2015). The ethical approval stated that written consent to participate in the study was not necessary. The study was retrospectively registered on December 7th, 2017, in the German Clinical Trials Register (DRKS00013184).

## Method

### Study design

**Fig. 1:**
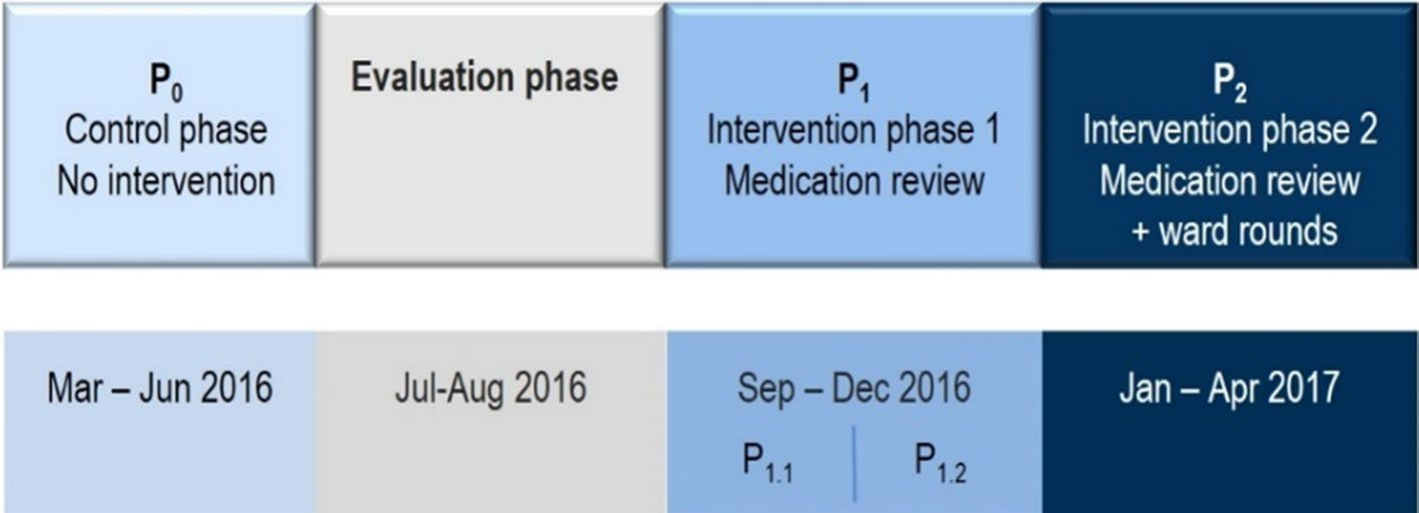
Study design; Intervention phase P_1_ was subdivided into P_1.1_ and P_1.2_. Each subdivision lasted 2 months. The PE rate in P_1.1_ was compared to the PE rate in P_1.2_ to detect possible learning effects of the physicians during study phase P_1_

The study was divided into four phases. One retrospective control phase (P_0_), one evaluation phase and two intervention phases (P_1_ and P_2_) were conducted, as shown in Fig. 1.

### P_0_—Control phase

During the control phase, the data were collected retrospectively without any intervention. For ethical reasons, the evaluation of the patients’ medical records took place after the patient in question had been discharged from the hospital (see below). The CP (DM) screened all medical records of included patients to identify PEs made on weekdays (Monday–Friday; national holidays excluded).

### Evaluation phase

All detected possible PEs meeting the defined criteria for a PE (see “Definition of prescribing errors”) were discussed with the chief physician (MT) of the Department of Anaesthesia. If the chief physician (MT) agreed, the PEs found by the CP (DM) were included. If no consensus could be reached, specific external experts were consulted. We had an external expert for each of the specific disciplines: anaesthesiology, intensive care medicine, internal medicine, nephrology, microbiology and neurology. Existing standards of care (SOP) were modified or new SOPs developed if necessary (e.g. new published guidelines or new findings). The retrospective design of the baseline period was intended to avoid ethical conflicts.

### P_1_—Intervention phase 1

Two CPs (NK&DM) were present on the ward Monday–Friday from 9 am to 1 pm. They reviewed the medical records, collected data, and provided information on drugs and internal standards of care. Information about the patients was received through the medication orders, laboratory findings and medical records. While reviewing the prescriptions, special attention was paid to adherence to the internal SOPs, dose adjustment according to organ function, documentation, indications, contraindications, interactions and the continuation of necessary long-term medication. PEs meeting the defined criteria were documented and discussed with the senior physician in charge of the ward. If the senior physician in charge agreed, a PE was included. All identified PEs were discussed once a week with all physicians on the ward for educational purposes. Furthermore, every physician in the department received an email from the CPs with the PEs from the prior week and their explanations to ensure continued learning.

In order to detect possible learning processes, P_1_ was subdivided into two subphases (P_1.1_ and P_1.2_; Fig. 1), each of which lasted 2 months. The comparison of the identified PEs (P_1.1_ vs. P_1.2_) enabled the detection of possible learning processes of the physicians throughout P_1_.

### P_2_—Intervention phase 2

One CP became a member of the daily ward round in addition to conducting the medical record review and the other activities introduced in P_1_; thereby, the CP received more information about the patients, such as any planned surgeries, therapeutic interventions and radiology and nuclear medicine findings. The CP provided important information about the detected PEs, which were discussed with the physicians during the rounds.

P_2_ was designed to determine if the additionally obtained information during the ward rounds had an influence on the impact of the CPs compared to their impact in P_1_.

### Setting

A controlled interventional study was conducted in an adult 12-bed surgical ICU in a tertiary-care hospital in Kassel, Germany.

### Inclusion and exclusion criteria

Patients aged 18 years and older were included in the study if they stayed in the ICU for ≥ 24 h (workdays only). If a discharged patient returned within 72 h, the patient remained in the study. Patients readmitted to the ICU after more than 72 h were considered new cases.

The medical charts of patients with incomplete documentation (e.g. no documentation for at least 24 h available) and those who were only admitted for monitoring or palliative patients were excluded.

### Outcomes

The primary outcome was the number of PEs detected that were related to the monitored medications.

The secondary outcomes were the number of PEs rated as potentially severe and the number of monitored days without systemic anti-infective therapy.

Furthermore, a descriptive analysis was made concerning the reasons for, acceptance of and measures resulting from the CPs’ interventions.

### Definition of prescribing errors

Medication errors can occur during the prescription, formulation, dispensing and administration of drugs. Because of the retrospective control phase of this study, only PEs were considered. Medication errors caused by nursing staff were excluded.

PEs meeting the criteria for medication errors based on the DokuPIK^1^ classification (listed in Table 1) were defined as PEs in this study. Unfounded deviations from the clinic-specific internal SOPs were also considered PEs. Before a PE identified by the CPs was included, a consensus between the CPs and the physicians was obligatory. If no consensus could be achieved, further expertise from various specialists was sought, as mentioned above. These requirements were necessary to ensure the clinical relevance of the study results. For every drug prescribed, the CPs evaluated whether the medication was correct or erroneous on every monitored patient-day. The identified PEs were related to the total number of monitored medications, in contrast to other studies in the ICU that related medication errors to the number of monitored patient days. If the same PE occurred on several days, it was included for each day.

**Table 1:** DokuPIK criteria on medication errors

Unnecessary drug
Drug indicated but not prescribed
Drug allergy or medical history not considered
Double prescription
Inappropriate or not most suitable drug formulation in terms of indication
Inappropriate or not most suitable drug in terms of indication
Prescription/Documentation incomplete/incorrect
Transcription error
Inappropriate administration (route) prescribed
Inappropriate administration (duration) prescribed
Failure to adjust dose for organ dysfunction
Inappropriate dose
Inappropriate administration interval
TDM not performed or neglected
Contraindication
Failure to discontinue relevant drugs preoperatively
Interaction

### Potentially severe prescribing errors

All identified PEs were discussed with the chief physician (MT) and rated as minor clinically relevant or potentially severe using the four judging criteria listed in Table 2.

**Table 2:** Judging criteria for potentially severe prescribing errors

May have increased the risk of mortality
May have resulted in organ damage
May have resulted in prolongation of the LOS
Increased costs > 100 €/day

If a PE was considered to lead to increased mortality, organ damage, prolongation of the length of stay (LOS) or increased costs (> 100€/day), it was considered a potentially severe PE. If necessary, external experts in the medical specialties relevant to the specific case (see “Evaluation phase”) were consulted to increase the quality and objectivity of the rating

### Qualitative analysis

A qualitative analysis of the PEs was undertaken by the CPs to determine the reasons for the pharmaceutical interventions, the acceptance of the interventions by the physicians and the resulting measures.

### Monitored days without systemic anti-infective therapy

**Fig. 2:**
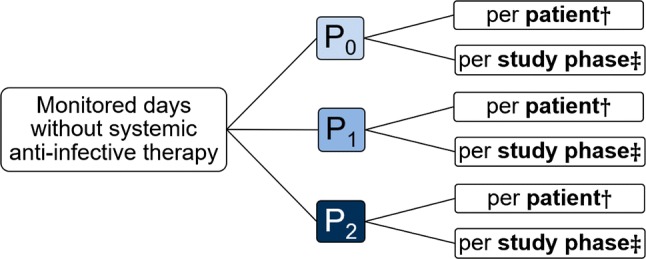
Monitored days without systemic anti-infective therapy; † Mann–Whitney U test; ‡ Fisher’s exact test. The total number of days without systemic anti-infective therapy was counted per patient. Additionally, for every study phase, the total number of days without systemic anti-infective therapy was evaluated. The total number of monitored days without systemic anti-infective therapy per study phase was then related to the total number of monitored patient days during the phase in question

To evaluate the impact of the CPs’ medication review on the usage of anti-infective drugs, we collected data on the monitored days without systemic anti-infective therapy (Fig. 2). Every monitored day without the prescription of a systemic antibiotic, antifungal agent and/or virostatic agent was counted.

### Calculation of the case numbers and statistical analysis

With an assumed PE rate of 2% as the baseline, the number of medications that needed to be monitored in each group was determined to be at least 2021 (a power of 80% and a type-I-error of 0.05) to detect a reduction of 50% [14]. The sample size was powered to detect differences in the incidence of PEs throughout the phases. Every monitored day the pharmacists counted all included medications that had been reviewed and all PEs that had been found.

Fishers’ exact test was used for binary values, and the Mann–Whitney U test was used for parametric values, as shown in Table 3. A *p* value < 0.05 was considered statistically significant. Statistical analysis was performed with IBM SPSS Statistics^®^ Versions 23&24.

**Table 3:** Overview statistical tests

Variables	Statistical test
Age	Mann–Whitney-U
Sex	Fisher’s Exact
SAPS II score	Mann–Whitney-U
Number of medications on the first day on ICU	Mann–Whitney-U
LOS all ICU at KKS	Mann–Whitney-U
LOS KKS	Mann–Whitney-U
Days of invasive mechanical ventilation	Mann–Whitney-U
Days of mechanical ventilation	Mann–Whitney-U
Renal replacement therapy	Fisher’s exact
Acute renal failure on admission to ICU	Fisher’s exact
Acute kidney damage for ≥ 3 days during ICU stay	Fisher’s exact
Hepatic insufficiency	Fisher’s exact
Prescribing errors	Fisher’s exact
Potentially severe prescribing errors	Fisher’s exact
Monitored days without SAIT per study phase	Fisher’s exact
Monitored days without SAIT per patient	Mann–Whitney-U

*KKS* Kassel hospital, *LOS* Length of stay, *SAIT* Systemic anti-infective therapy

## Results

### Demographic and clinical characteristics of the study population

**Fig. 3:**
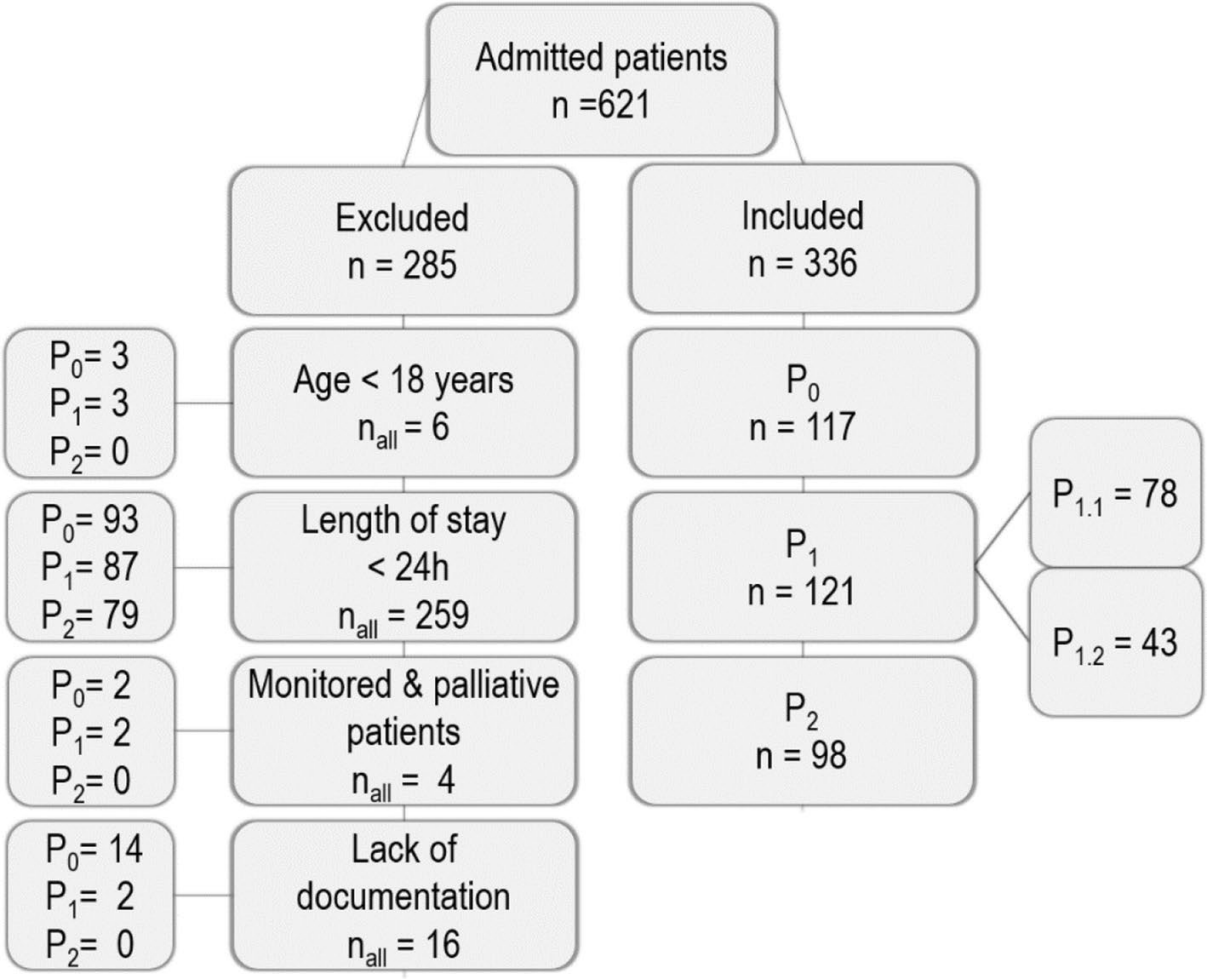
Participant flow chart

In the study period, 621 patients were admitted to the ICU, of whom 336 were included (Fig. 3). The characteristics of the study population and the data for the monitored days without systemic anti-infective therapy are listed in Tables 4 and 7.

**Table 4:** Demographic and clinical characteristics of the study population

Characteristics	P_0_ (n = 117)	P_1_ (n = 121)	P_2_ (n = 98)
Age [median (25th; 75th quartiles)]^a^	64.0 (53; 76.5)	68.0 (59.0; 76.0)	68.0 (56.75;77.0)
Male [n (%)]^b^	70 (59.8)	80 (66.1)	66 (67.3)
SAPS II [median (25th; 75th quartiles)]^a^	37.5 (27.25; 52.0)	39.0 (28.0; 50.5)	40.0 (29.75; 48.25)
Number of medications on the first day on ICU [median (25th; 75th quartiles)]^a^	13.0 (10.0; 17.0)	14.0 (11.0; 16.0)	13.0 (10.0; 17.0)
LOS all ICU at KKS [days, median (25th; 75th quartiles)]^a^	10.0 (5.5; 21.0)	9.0 (4.0; 17.0)	11.0 (5.0; 24.25)
LOS KKS [days, median (25th; 75th quartiles)]^a^	26.0 (18.0; 41.0)	29.0 (16.5; 41.5)	32.0 (19.0; 55.0)^*^
Days of invasive mechanical ventilation [median (25th; 75th quartiles)]^a^	4.0 (2.0; 11.0)	2.0 (0.0; 7.0)^*^	4.5 (1.0; 13.5)^*^
Days of mechanical ventilation [median (25th; 75th quartiles)]^a^	6.0 (2.0; 13.0)	3.0 (1.0; 12.0)^*^	7.0 (2.0; 15.0)^*^
Renal replacement therapy [n (%)]^b^	22 (18.8)	29 (24.0)	23 (23.5)
Acute renal failure on admission to ICU [n (%)]^b^	24 (20.5)	20 (16.5)	10 (10.2)
Acute renal failure for ≥ 3 days during ICU stay [n (%)]^b^	22 (18.8)	24 (19.8)	17 (17.3)
Hepatic insufficiency (Bilirubin > 5 mg/dL or ALAT > 100 U/L [n (%)]^b^	39 (33.3)	44 (36.4)	35 (35.7)

Statistical tests: ^a^Mann–Whitney-U Test; ^b^Fisher’s Exact Test; ^*^p-value < .05 (P_1_ vs P_0_ respective P_2_ vs. P_1_)

*KKS* Kassel hospital, *LOS* Length of stay

### Prescribing errors

The percentages of PEs in phase P_1_ (5.13%) and P_2_ (3.25%) were significantly lower (*p* < 0.001) than that in phase P_0_ (14.12%) (see Table 5). While in P_0_, 1986 PEs per 1000 monitored patient days occurred, this rate was reduced to 772 in P_1_ and 479 in P_2_ (Table 5).

**Table 5:**
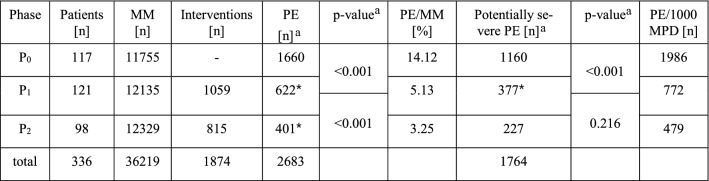
Results of the study phases

*MM* monitored medications, *PE* prescribing errors, *MPD* monitored patient-days, Statistical test: ^a^Fisher’s Exact Test, ^*^ = *p* < 0.001

Regarding the learning process, compared to P_1.1_, fewer PEs occurred in P_1.2_ [274 PEs vs. 348 PEs (*p* < 0.001)].

### Potentially severe prescribing errors

The total rates of potentially severe PEs related to the monitored medications were found to be 9.87% in P_0_, 3.11% in P_1_, and 1.84% in P_2_ (Table 5).

### Descriptive analysis

The CPs performed 1874 interventions during P_1_ and P_2_. Agreement with the physicians was reached in 77.8% (P_1_) and 80.7% (P_2_) of all interventions.

Most of the PEs in the study were the result of “drug indicated but not prescribed”, as shown in Table 6.

**Table 6:** Top 5 Medication errors

	P_0_ (nMM = 11,755)	P_1_ (nMM = 12,135)	P_2_ (nMM = 12,329)
1	Drug indicated but not prescribed nPE = 361	Drug indicated but not prescribed nPE = 126	Drug indicated but not prescribed nPE = 82
2	Inappropriate administration route or handling prescribed nPE = 240	TDM not performed or neglected nPE = 79	Inappropriate dose nPE = 59
3	Failure to adjust dose for organ dysfunction nPE = 196	Documentation incorrect nPE = 74	Documentation incorrect nPE = 56
4	Discontinuation of long-term medication nPE = 183	Inappropriate dose nPE = 70	TDM not performed or neglected nPE = 53
5	Inappropriate dose nPE = 177	Unnecessary drug nPE = 66	Unnecessary drug nPE = 37

*nMM* Number of monitored medications, *nPE* Number of monitored prescribing errors, *TDM* therapeutic drug monitoring

### Monitored days without systemic anti-infective therapy

Regarding the number of monitored days without systemic anti-infective therapy for each patient, medians of 1.0 were detected in all three phases (Table 7). The 75th percentiles were 3.0 days (P_0_, P_1_) and 4.0 days (P_2_). Relating the number of all monitored days without systemic anti-infective therapy to the total number of monitored patient days, significantly (*p* < 0.001; Fisher’s exact test) more days without systemic anti-infective therapy occurred in P_1_ than in P_0_ (Table 7).

**Table 7:**
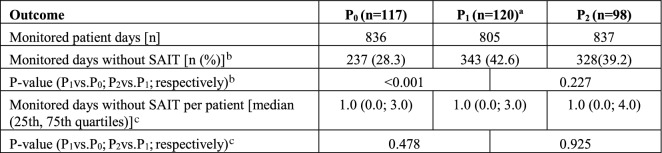
Results of outcome days without systemic anti-infective therapy

*SAIT* systemic anti-infective therapy

Statistical tests: ^a^data of one case missing, ^b^Fisher’s exact test, ^c^Mann–Whitney *U* test

## Discussion

### Demographic and clinical characteristics of the study population

All patient groups were homogenous regarding the ICU length of stay (LOS), organ failure and SAPS II. The patients’ severity of illness in the three phases appear to be comparable. Differences were found in the number of days of invasive mechanical ventilation and the number of days of mechanical ventilation (Table 4). The differences are irrelevant concerning the severity of illness of the patients because other parameters, such as the SAPS II and the ICU LOS, were comparable.

### Prescribing errors

Throughout the baseline, 1660 PEs were identified in 11.755 monitored medications. The detected reduction in PEs [622 PEs in 12.135 monitored medications (P_1_) and 401 PEs in 12.329 monitored medications (P_2_)] supports the assumption that a CP’s medication review results in a significant reduction in the number of PEs in relation to the number of PEs that occurred in a control group. An additional involvement of the CP during the ward rounds resulted in a further reduction in PEs, as shown by the findings in P_2_ (479 PEs/1000 monitored patient days). Compared to P_1_, the rate of PEs was decreased from 5.13% to 3.25% by the implementation of CP participation in ward rounds (on workdays). As expected, significantly fewer PEs occurred in P_1.2_ than in P_1.1_ (274 PEs vs. 348 PEs), showing a learning process throughout the intervention phase P_1_. This learning effect reflects the result of better teamwork and the addition of the CP to the ICU team, which led to a longitudinal interdisciplinary education process for all participating professional groups.

The rate of 1986 PEs/1000 monitored patient days and 1388 potentially severe PEs/1000 monitored patient days differs from the findings of Klopotowska et al. (190.5 medication errors/1000 monitored patient days) and Kaushal et al. (29 serious medication errors/1000 monitored patient days) [1, 8]. This might be attributed to different definitions, methods of counting medication errors and study settings. Unlike in other studies, all PEs in the present study always required the approval of a physician to be included and were counted at each occurrence. The major reason for the daily count was the instability of the condition of intensive care patients. A proper medication or dosage on one day may need adaption or may even be harmful or contraindicated on the next day (e.g., antibiotic dosage), rendering the daily monitoring of the patients and their medications even more important. To generate accurate and conclusive results, the PEs and the monitored medications had to be counted in the same way, i.e. daily.

The PEs were related to the monitored medications. This made it possible to decide whether there was an error for every drug prescribed. The resulting PE rate was more precise than the relation of PEs to patient days, especially with regard to intensive care patients. The differing needs and complexity of the patients meant that one “monitored patient-day” might pertain to five, eight or even 24 monitored medications. In our calculation model, these differences were considered, and a PE rate could be determined.

The significant reduction in the rate of potentially severe PEs from 9.87% in P_0_ to 3.11% in P_1_ and the further reduction to 1.84% in P_2_ indicates a positive influence of the CPs’ interventions on medication safety. PE classifications determined by a physician as in our study, could be different from classifications determined by pharmacists, as in the study by Klopotowska [1]. It has been shown that physicians tend to rate the impact of pharmacy services more favourably than do pharmacists themselves [15]. However, by requiring the physicians’ approval, a clinically relevant study result was guaranteed.

The most common cause of the interventions was the need for an additional drug in all three phases. This was mostly owing to deviations from the SOPs. By communicating the internal SOPs, pharmacists can contribute to the improvement of guideline adherence in the ICU. Drugs that should have been prescribed were initiated so that there was no great impact on the cost. Furthermore, proper stress ulcer prophylaxis and correct use of laxatives might contribute to preventing follow-up costs and increased LOS for the treatment of, e.g., gastric ulcerations or ileus [16]. The number of PEs resulting from “drug indicated, but not prescribed” decreased throughout the study phases. The CP has an impact on this source of PEs, but it remains relevant.

Regarding the top 5 PEs in the intervention phases, CPs made important contributions to reducing dosing issues and TDM, increasing guideline compliance, deprescribing and the clarification of medication orders.

The acceptance rates of the CPs’ recommendations of 77.8% (P_1_) and 80.7% (P_2_) are comparable to those in other international studies (74–87%), while some other studies reached a consensus rate of more than 90% [1, 3, 7, 17, 18]. The high quality of the CPs’ interventions is shown by the acceptance rate, which could be increased by even more interdisciplinary cooperation, confering even more importance on interdisciplinary communication. The cases in which the physician disagreed with the pharmacists’ interventions might partly be attributed to individual patient needs that were not adequately covered by the SOPs.

### Monitored days without systemic anti-infective therapy

A positive influence on the number of monitored days without systemic anti-infective therapy was demonstrated. The total number of days without systemic anti-infective therapy was significantly higher in P_1_ than in P_0_. In a previous study, Weber et al. achieved a significantly shorter duration of antibiotic therapy through pharmaceutical interventions on a surgical ward [19]. The present study, however, was not powered to detect an effect on the use of anti-infective agents. Different clinical settings, patient conditions and indications for medication make it difficult to compare the anti-infective therapies and the results. However, this positive trend should be the subject of further investigations in the context of antibiotic stewardship. There is no need for a specialized CP to achieve an improvement in medication safety. In particular, if no patient data management system is available, the impact of pharmacists’ interventions might be even greater. In our opinion, this study should embolden CPs to offer their expertise in a multidisciplinary setting.

### Limitations

The chosen study design limits the present study. During the baseline period, the PEs were detected, discussed and rated retrospectively for ethical reasons. It would have been ethically not acceptable that a detected PE would not have led to an intervention (e.g. wrong antibiotic in a septic patient). In a prospective setting, any detected PEs have to be corrected immediately for ethical and legal issues, leading to bias, and the effect of physician learning cannot be ruled out. In the intervention period, the detected PEs had to be discussed prospectively with the senior physician in charge of the ward as part of the daily routine. Prospectively, it is more difficult to predict the consequences of a detected PE for the individual patient. Retrospectively, it is difficult not to take the therapeutic outcome of the treatment into account. Bias must be considered when judging the PEs, e.g., the choice of antibiotic agents. However, when assessing PEs, there will always be ethical issues prohibiting the use of prospective control groups.

## Conclusion

Clinical pharmacists’ interventions led to a significant reduction in the PE rate in a German ICU, contributing to a safer medication process. Daily on-ward participation by CPs reduced the incidence of potentially severe PEs. Positive trends concerning the targeted and proper use of anti-infective drugs need further research. Regarding the positive international findings on critical care pharmacy, our findings are in line with those of other international studies, leading us to strongly recommend a broad introduction of CPs into German ICUs.

## Electronic supplementary material

Below is the link to the electronic supplementary material.

Supplementary material 1 (DOCX 15 kb)

## Abbreviations

CP: Clinical pharmacist
DokuPIK: Database used in the hospital for the documentation of pharmacists’ interventions (Dokumentation pharmazeutischer Interventionen im Krankenhaus)
ICU: Intensive care unit
KKS: Kassel hospital
LOS: Length of stay
MM: Monitored medications
MPD: Monitored patient-day(s)
nMM: Number of monitored medications
nPE: Number of monitored prescribing errors
P_0_: Control phase
P_1_: Intervention phase 1
P_1.1_: Subdivision 1 of intervention phase 1
P_1.2_: Subdivision 2 of intervention phase 1
P_2_: Intervention phase 2
PE: Prescribing error
SAIT: Systemic anti-infective therapy
SAPS II: Simplified acute physiology score II
SOP: Standard operating procedure
TDM: Therapeutic drug monitoring

## Footnotes

1 The German Association of Hospital Pharmacists’ (ADKA) database for the documentation of pharmaceutical interventions in hospitals.
