# Supplementary material for: A new approach on assessing clinical pharmacists’ impact on prescribing errors in a surgical intensive care unit
Source: Int J Clin Pharm. 2019 Jul 22;41(5):1184–92. doi: 10.1007/s11096-019-00874-8 (PMC6800837; doi:10.1007/s11096-019-00874-8)
Supplement: Supplementary file 1 — Supplementary material 1 (DOCX 15 kb) [file 11096_2019_874_MOESM1_ESM.docx]

Appendix

Decision process for prescribing errors

If a PE according to DokuPIK or the product characteristics occurred that stood in contradiction to a SOP, we decided in favour of the SOP to guarantee clinical relevance by including the current state of research.

| Issue | DokuPIK –  product characteristics | SOP |
| --- | --- | --- |
| Continuous application of antibiotics | Prescribing error – off label use | No prescribing error – current state of research |
| No loading dose given before initiation of continuous application of antibiotics | No prescribing error | Prescribing error – current state of research |
| Application of Levosimendane <GFR 30 mL/min | Prescribing error – contraindication | No prescribing error – if lack of alternatives for therapy |
| Sucralfate | Prescribing error – max. 6 g/day | 3x3 g per day[20] |

Examples for potentially severe prescribing errors

Examples of PEs rated as potentially severe are listed in the table below.

| **Prescribing error** | **Potential consequences** |
| --- | --- |
| No antibiotic although indicated | Sepsis, death |
| Wrong antibiotic despite microbial test | Sepsis, death |
| Antibiotic dose too low | Antimicrobial resistance, sepsis, death |
| No proper thromboembolism prophylaxis | Stroke, pulmonary embolism, …, death |
| No proper stress-ulcer prophylaxis | Gastric-ulcer, risk of bleeding, LOS ↑ |

Overview all prescribing errors detected

| **Prescribing error** | **P₀ [n]** | **P₁ [n]** | **P₂ [n]** | **Total [n]** |
| --- | --- | --- | --- | --- |
| Drug indicated but not prescribed | 361 | 126 | 82 | 570 |
| Inappropriate dose | 177 | 70 | 59 | 307 |
| Inappropriate administration route or handling prescribed | 240 | 38 | 4 | 282 |
| Failure to adjust dose for organ dysfunction | 196 | 55 | 27 | 278 |
| Documentation incorrect | 112 | 74 | 56 | 242 |
| Discontinuation of long-term medication | 183 | 35 | 22 | 239 |
| TDM not performed or neglected | 97 | 79 | 53 | 228 |
| Unnecessary drug | 113 | 66 | 37 | 216 |
| Inappropriate drug | 75 | 14 | 4 | 93 |
| Wrong dosing interval | 39 | 16 | 13 | 68 |
| Duplication | 11 | 7 | 15 | 33 |
| Interaction | 17 | 9 | 1 | 27 |
| Incompatibility or incorrect preparation/reconstitution prescribed | 4 | 10 | 11 | 25 |
| Administration (duration) | 12 | 5 | 3 | 20 |
| Unnecessary laboratory test | 7 | 4 | 5 | 16 |
| Contraindication | 4 | 2 | 5 | 11 |
| Inappropriate drug formulation prescribed | 9 | 0 | 2 | 11 |
| Inappropriate duration of drug therapy | 0 | 9 | 1 | 10 |
| Administration (I.V. to oral switch) | 0 | 3 | 0 | 3 |
| Drug allergy | 3 | 0 | 0 | 3 |
| No discontinuation of medication needing discontinuation before surgical procedure | 0 | 0 | 1 | 1 |
| Total | 1660 | 622 | 401 | 2683 |
